# A case report of rectal schwannoma treated with laparoscopic proctectomy

**DOI:** 10.1097/MD.0000000000009866

**Published:** 2018-02-16

**Authors:** Kai Zhang, Shanshan Qu, Junan Li, Ye Cheng, Jian Shi, Tongjun Liu

**Affiliations:** a Department of General Surgery; b Department of Pathology; c Department of Endoscopy, The Second Hospital of Jilin University, Changchun, Jilin.; d Department of Neurosurgery, Xuanwu Hospital, Capital Medical University, Beijing, China.

**Keywords:** immunohistochemical staining, laparoscopic surgery, rectal neoplasm, schwannoma

## Abstract

**Rationale:**

Schwannomas of gastrointestinal tracts are rare and difficult to detect preoperatively because of negative results of endoscopic and imaging examinations. Here, we reported a case of rectal schwannoma, which was diagnosed by immunohistochemical staining after laparoscopic protectomy.

**Patient concerns:**

A 61-year-old woman complained of a 1-month history of difficulty in defecation and irregularly abdominal discomfort during her physical checkup in our hospital.

**Diagnoses:**

Immunohistochemical staining results after laparoscopic protectomy revealed a strong positive reaction for S-100 protein. Therefore, rectal schwannoma was confirmed.

**Interventions:**

Treatment with laparoscopic protectomy was given.

**Outcomes:**

Symptoms resolved completely after 12 days of the surgery, and was regular followed-up in outpatient clinic.

**Lessons:**

Schwannomas are difficult to identify preoperatively, and immunohistochemical staining for S-100 protein is an effective method to diagnose it.

## 1 Introduction

Schwannnoma is a tumor originating from the schwann cells, which forms the nerve sheaths. Schwann cells commonly grows along the peripheral nerves of the head and cervix, so schwannomas of gastrointestinal tracts are extremely rare.^[1]^ Laparoscopic surgery has been broadly applied to many colorectal diseases. It has been considered as a minimally invasive treatment for colorectal diseases.^[2]^ Here, we present a case of rectal schwannoma in a 61-year-old woman treated by laparoscopic surgery.

## 2 Case Report

This study was approved by the Ethics Committee and institutional review board of the Second Hospital of Jilin University, Changchun, China. Written informed consent was obtained from the patient for publication of this report.

**Figure 1:**
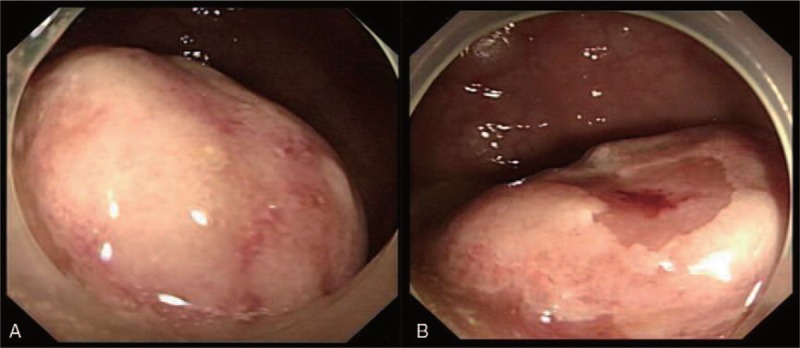
Semipedunculated polypoid lesion (4 cm) of the rectum was revealed by colonoscopy.

**Figure 2:**
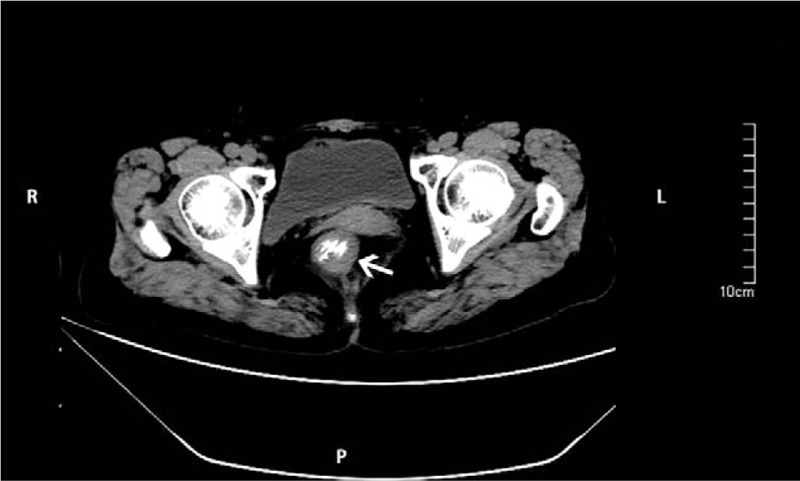
Abdominal CT findings. A polypoid lesion with homogeneous low attenuation inside was revealed in the rectum with adjacent wall thickening (white arrow).

A 61-year-old woman complained of a 1-month history of difficulty in defecation and frequently abdominal irregular discomfort during her physical checkup in our hospital. She had a 10-year history of well-controlled hypertension in her medical history. Physical examination showed no remarkable signs on abdomen but a firm mass with intact mucosa at 12 o’clock of the rectal wall detected via digital rectal examination. Colonoscopy revealed a tumorous lesion, which measured 4 cm in diameter, 6 cm from the anal verge, located in the rectum. The surface of the mucosa covering the lesion was intact (Fig. 1). Abdominal computed tomography (CT) revealed thickening of the anterior rectum (Fig. 2). There was no enlarged lymph node being identified. Considering the possibility of malignant tumor, removal of tumor was performed by laparoscopic surgery.

**Figure 3:**
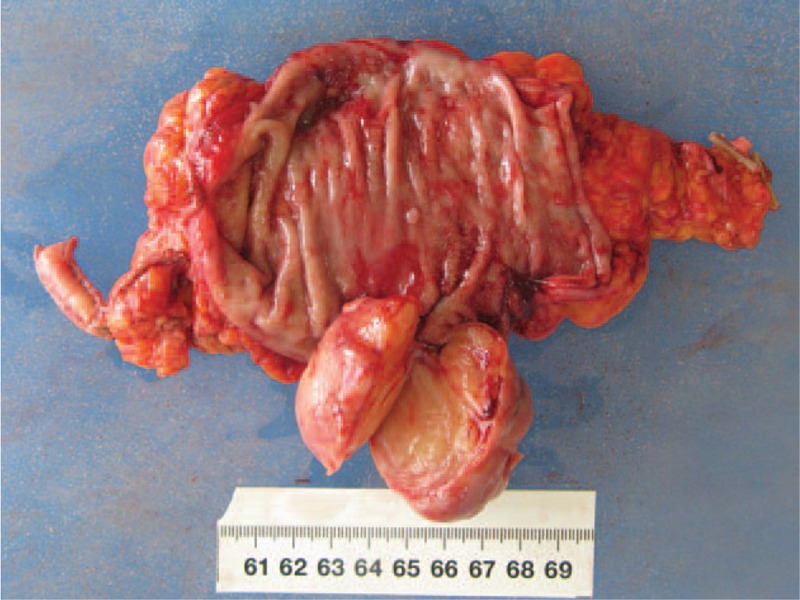
Gross findings: 4.9 cm × 4.8 cm polypoid growth.

**Figure 4:**
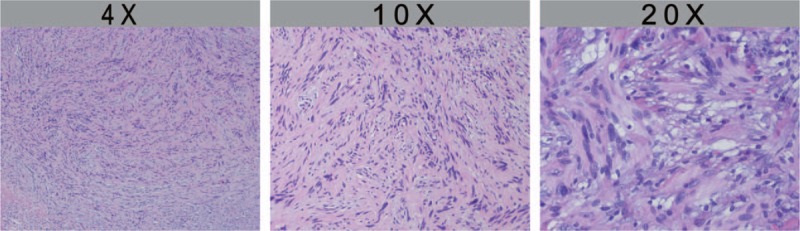
HE staining of mass showing spindle cell aggregate (4X, 10X, 20X).

**Figure 5:**
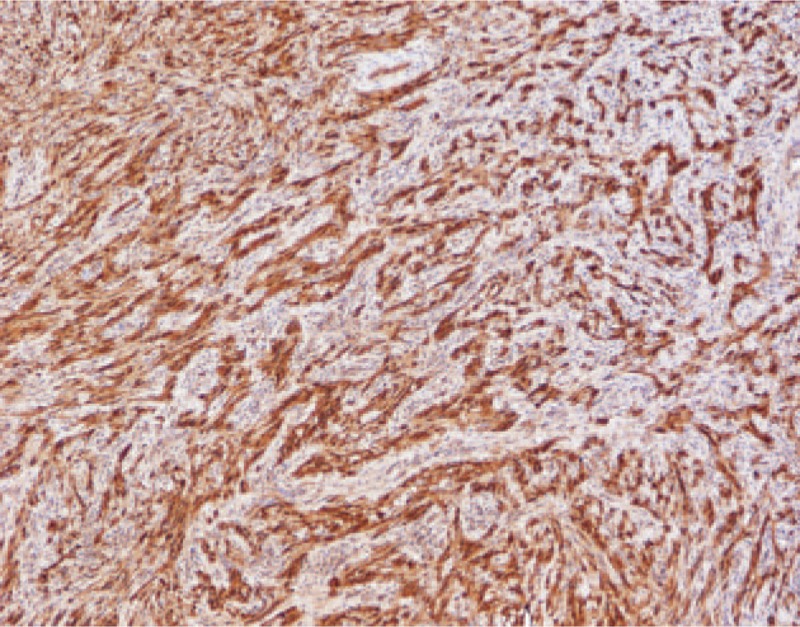
Strong immunoreactivity of tumor cells to S-100 protein.

Postoperatively, the tumor, about 4 × 5 cm in size, was covered by normal colonic mucosa of the rectum. The tumor was white-yellow in color, and the texture was firm and fibrotic (Fig. 3). Histopathological findings by hematoxylin–eosin staining of the resected specimen revealed spindle-like tumor cells with zonally variable cellularity (Fig. 4), and the tumor was comprised of a proliferation of spindle cells, which were arranged in nodular, fascicular, or vague palisading patterns. Tumor cells had eosinophilic, fibrillary cytoplasm, and elongated or pointed nuclei. No atypical mitotic or cellular atypism signs were found. Immunohistochemical staining results for S-100 protein revealed a strong positive reaction (Fig. 5), but were negative for CD117, α-smooth muscle actin, and desmin. Ki-67 labeling was demonstrated in fewer than 5% of tumor cells. There was no evidence of metastasis by the identification of 14 lymph nodes. On the basis of the above findings, we diagnosed it as benign rectal schwannoma. The patient was discharged from our hospital after a smooth recovery at postoperative day 12.

There were no signs of recurrence in the 3 months after the treatment. However, a long-term clinical follow-up should be given to make sure the uncertain prognosis.

## 3 Discussion

Schwannoma was first described in 1910. With the development of newer immunohistochemical staining technique in recent years, there has been an increase in reports of mesenchymal tumors. However, primary schwannomas of the colon and rectum are still difficult to discover preoperatively and are extremely rare.^[3]^ Because of the rare number of cases, the features of this tumor are not presented fully.

Gastrointestinal schwannomas generally present asymptomic masses or nonspecific symptoms such as rectal pain, bleeding, and tenesmus.^[3–5]^ Image examination including transrectal ultrasound, CT, barium enema, and colonoscopy are applied in the rectal tumors to measure tumorous margin, size, depth of tumorous penetration, relationships with adjacent structures, and the presence of metastases. However, there was no definite criteria of image to diagnose the rectal schwannoma.^[6]^ Colonoscopy could conduct a biopsy, but it is not suitable for a submucosal lesion and may not confirm benign from malignant tumor because of the restricted specimens. Hence, biopsy should be multiple sampling and accurate enough to reduce the false negative results of pathology. In this case, a rectal tumor with smooth surface was clearly observed by colonoscopy and was evaluated by abdominal CT in the meantime. Biopsy was taken; however, preoperative definite diagnosis was uncertain and malignancy could not be excluded completely because of the negative pathological results of the biopsy. Surgical resection was conducted. In this case, laparoscopic radical resection with lymphadenectomy was conducted instead of partial resection, the standard treatment for schwannomas of the digestive tract, because of the possibility of the possibility of tumor malignancy.

Literally, histopathological observations by hematoxylin–eosin staining of tumor tissue have shown that schwannomas are composed of elongated bipolar spindle cells with zonally variable cellularity and focally prominent nuclear palisading pattern.^[7]^ In immunohistochemical level, schwannoma cells are immunoreactive to S-100 protein, but are negative for smooth muscle actin, CD117, cytokeratin (CK), and desmin.^[8]^ Furthermore, a diagnosis of tumor malignancy is based on the status of the Ki-67 labeling index (MIB-1), whereby a highly reproducible immunopositivity for MIB-1 is considered as an indicator of malignancy.^[4]^ In this case, immunohistochemical findings include these criteria. Thus, we diagnosed it as benign schwannoma.

Schwannoma is a kind of benign tumor, the survival rate of it approaches to 100%.^[9]^ However, relapse is not uncommon. Development of cysts relapses up to 15% with the development of disease, approximately 9% to 45% of rectal malignant tumors are commonly solid than cystic, which has a recurrence rates of 45%, and its 5-year survival rate is merely about 8% to 17%.^[10]^ In our case, no evidence of aggressive behavior was found based on 3-month postoperative follow-up information, which indicated that benign schwannomas have a good prognosis. However, a long-term clinical follow-up should be given to monitor the possible malignancy.

In conclusion, rectal schwannomas are extremely rare and only few cases have been reported; immunohistochemical staining for S-100 protein is an effective method for differential diagnosis; and schwannomas are frequently diagnosed as benign tumors and carry a good prognosis.
